# Transplantation of Neural Stem Cells Loaded in an IGF‐1 Bioactive Supramolecular Nanofiber Hydrogel for the Effective Treatment of Spinal Cord Injury

**DOI:** 10.1002/advs.202306577

**Published:** 2024-03-05

**Authors:** Peiwen Song, Tianyu Han, Zuomeng Wu, Huang Fang, Yunlei Liu, Wang Ying, Xianwen Wang, Cailiang Shen

**Affiliations:** ^1^ Department of Orthopedics (Spinal Surgery) Laboratory of Spinal and Spinal Cord Injury Regeneration and Repair The First Affiliated Hospital of Anhui Medical University Hefei 230032 China; ^2^ Anhui Province Research Center for the Clinical Application of Medical Technology The First Affiliated Hospital of Anhui Medical University Hefei 230032 China; ^3^ Department of Orthopedics (Spinal Surgery) The First Affiliated Hospital of USTC Hefei 230032 China; ^4^ Department of Clinical Laboratory The First Affiliated Hospital of Anhui Medical University Hefei 230032 China; ^5^ Department of Medical Imaging The First Affiliated Hospital of Anhui Medical University Hefei 230032 China; ^6^ School of Biomedical Engineering Research and Engineering Center of Biomedical Materials Anhui Provincial Institute of Translational Medicine Anhui Medical University Hefei 230032 P. R. China

**Keywords:** extracellular vesicles, hydrogel, insulin‐like growth factor‐1, neural stem cells, spinal cord injury

## Abstract

Spinal cord injury (SCI) leads to massive cell death, disruption, and demyelination of axons, resulting in permanent motor and sensory dysfunctions. Stem cell transplantation is a promising therapy for SCI. However, owing to the poor microenvironment that develops following SCI, the bioactivities of these grafted stem cells are limited. Cell implantation combined with biomaterial therapies is widely studied for the development of tissue engineering technology. Herein, an insulin‐like growth factor‐1 (IGF‐1)‐bioactive supramolecular nanofiber hydrogel (IGF‐1 gel) is synthesized that can activate IGF‐1 downstream signaling, prevent the apoptosis of neural stem cells (NSCs), improve their proliferation, and induce their differentiation into neurons and oligodendrocytes. Moreover, implantation of NSCs carried out with IGF‐1 gels promotes neurite outgrowth and myelin sheath regeneration at lesion sites following SCI. In addition, IGF‐1 gels can enrich extracellular vesicles (EVs) derived from NSCs or from nerve cells differentiated from these NSCs via miRNAs related to axonal regeneration and remyelination, even in an inflammatory environment. These EVs are taken up by autologous endogenous NSCs and regulate their differentiation. This study provides adequate evidence that combined treatment with NSCs and IGF‐1 gels is a potential therapeutic strategy for treating SCI.

## Graphical abstract

In present study, an IGF‐1‐bioactive supramolecular nanofiber hydrogel is synthesized to promote neurite outgrowth and myelin sheath regeneration following SCI by improving NSC proliferation, preventing cell apoptosis, promoting differentiation of the grafted NSCs into neurons and oligodendrocytes, and enriching the beneficial cargoes within their released EVs which formed long‐distance cell‒cell contacts with autologous endogenous NSCs.

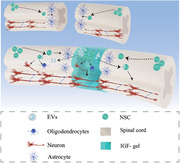

## 1 Introduction

Spinal cord injury (SCI) is caused by primary mechanical force on the spinal cord that results in permanent motor and sensory dysfunction.^[^ ^1^ ^]^ In response to primary spinal cord damage, posttraumatic local inflammation is activated, leading to the overproduction of inflammatory cytokines, axonal inhibitory factors, and radical oxygen species (ROS), ultimately resulting in massive cell death, neuronal apoptosis, and disruption and demyelination of axons. Currently, there is no effective treatment for promoting axonal and neural regeneration or replacing lost nerve cells, but a promising strategy is stem cell transplantation.^[^ ^2^ ^]^ Neural stem cells (NSCs) are a group of self‐renewing and multipotent stem cells that are present in the central nervous system (CNS).^[^ ^3^ ^]^ Following neurotrauma, they can be activated and migrate to lesion sites, contributing to neurogenesis, regeneration, and remyelination of axons and promoting the self‐recovery of neurological function following CNS injury.^[^ ^3a,b^ ^]^ Therefore, studies have attempted to transplant NSCs to replace damaged nerve cells after neurotrauma.^[^ ^3^, ^4^ ^]^ However, due to the poor and inhibitory microenvironment that develops following SCI, the therapeutic application of NSCs has faced a series of challenges, including a high rate of apoptosis of exogenously transplanted NSCs and a lack of neural and oligodendroglial differentiation, leading to most NSCs differentiating into astrocytes, which causes overgrowth of astrocytic scars.^[^ ^3^, ^5^ ^]^ In addition, emerging evidence has shown that the paracrine activity of transplanted stem cells is mostly responsible for positive biological outcomes.^[^ ^6^ ^]^ Extracellular vesicles (EVs) are membrane‐delimited particles secreted by nearly all cell types.^[^ ^7^ ^]^ They carry diverse bioactive cargoes (such as lipids, miRNAs, proteins, and nucleic acids) and are crucial for regulating physiological and pathological processes in the CNS.^[^ ^7^, ^8^ ^]^ However, the bioactivity and biological cargo of EVs can be modified by external stimuli and the surrounding microenvironment.^[^ ^9^ ^]^ The paracrine capacity of grafted cells may be negatively impacted by their poor environment after SCI, and they may discharge harmful cargoes. Therefore, providing a favorable environment to overcome these challenges will lead to tremendous progress in cell transplantation therapies for SCI.

Advances in biomaterials science have led to the development of innovative strategies for the transplantation of NSCs.^[^ ^10^ ^]^ Among all biomaterials, hydrogels have been favored by researchers because of the following advantages.^[^ ^11^ ^]^ First, hydrogels are usually nontoxic, and their compliant physical properties allow them to adapt to any irregular lesion site. Second, hydrogels could provide a 3D culture environment that not only prevents cell dispersion and promotes the growth of implanted cells but also separates these cells from the inhibitory environment. Finally, the mechanical properties can be modified to match those of the tissues of the spinal cord. Many studies have implanted hydrogels combined with stem cells into lesion sites following SCI and have achieved promising outcomes.^[^ ^5^, ^11^, ^12^ ^]^ Although hydrogels can provide a favorable environment for the survival, proliferation, and differentiation of grafted cells, a lack of nutritional factors limits the biological capacity of these exogenous cells.^[^ ^10^, ^13^ ^]^ Therefore, various nutritional agents are transplanted in combination with stem cells or hydrogels in an attempt to compensate for the deficiency in nutritional factors at injury lesion sites.^[^ ^14^ ^]^

Insulin‐like growth factor‐1 (IGF‐1) is a growth hormone that plays a critical role in multiple cellular processes, including neurogenesis.^[^ ^15^ ^]^ Studies have also supported the essential role of IGF‐1 in NSC self‐renewal, proliferation, differentiation, and neurogenesis.^[^ ^16^ ^]^ IGF‐1 has therefore been shown to be effective in treating neurotrauma, including SCI.^[^ ^17^ ^]^ However, the direct effects of IGF are similar to those of other conventional growth factors, which have short half‐lives, poor tissue retention, and difficulty maintaining bioactivities in vivo, limiting their bioactivities.^[^ ^18^ ^]^ Recent studies have designed peptides containing functional amino acid sequences to mimic the biological functions of targeted growth factors. In addition, these designed peptides can self‐assemble into different types of nanomaterials via noncovalent interactions to form nanostructures or hydrogels.^[^ ^18^, ^19^ ^]^ For example, Stupp et al. designed QK peptides that could mimic the biological function of vascular endothelial growth factor (VEGF).^[^ ^19a^ ^]^ Furthermore, compared to that of conventional growth factors, the half‐life of these synthetic peptides could be significantly prolonged when the peptides were assembled with nanomaterials to form supramolecular nanofibers. When these synthetic peptides are bound to nanomaterials, they exhibit improved tissue retention, allowing them to exert their biological activities continuously and stably in vivo.

**Scheme 1:**
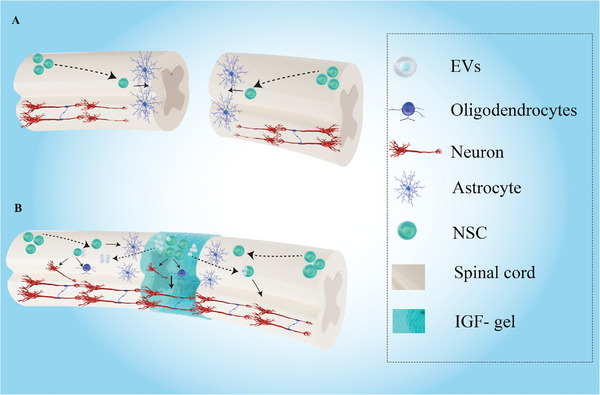
Schematic diagram of IGF‐1 gels carrying NSCs for enhancing the regeneration of neurons and myelin sheaths after SCI. A) Endogenous NSCs become active and move toward the lesion site after SCI. However, due to the adverse microenvironment at the lesion site, most of these NSCs differentiate into astrocytes, thereby forming an astrocytic scar, which inhibits the regeneration of neurons and neurite outgrowth. B) Implantation of IGF‐1 gels can provide a favorable environment for the survival, proliferation, and differentiation of grafted NSCs, causing them to form a “relay station” to connect neuronal fibers originating from intrinsic neurons. In addition, these grafted NSCs can secrete EVs, which are taken up by endogenous NSCs. By regulating the differentiation of these endogenous NSCs, EVs enhance neurite outgrowth and promote myelin sheath regeneration following SCI.

To carry NSCs to lesion sites following SCI and investigate whether biomaterials affect the bioeffects of EVs derived from these NSCs, a supramolecular nanofiber hydrogel was synthesized by attaching a self‐assembling peptide to the N‐terminus of the IGF‐1 C‐region, the IGF‐1 mimetic peptide hydrogel, which was able to activate the IGF and phosphatidylinositol 3 kinase/protein kinase B (PI3K/Akt) signaling pathways. According to the test of the biocompatibility and biodegradability of the IGF‐1 gel, no systemic toxicity was noted in vitro or in vivo, and the synthesized IGF‐gel could be gradually degraded to form a “bridge” across the lesion site to connect the rostral and caudal spinal cord. The synthesized IGF‐1 gel promoted the survival, proliferation, and differentiation of NSCs into neurons and oligodendrocytes. The implantation of IGF‐1 gels harboring NSCs significantly enhanced neurite outgrowth and myelin sheath regeneration within lesion sites, resulting in improved neurological recovery following SCI. In addition, detecting the expression of miRNAs within EVs derived from NSCs revealed that the IGF‐1 gel could enrich EVs shed by NSCs via miRNAs related to axonal regeneration and remyelination, especially in an inflammatory environment. Moreover, by investigating the biological activities of these EVs, it was shown that the IGF‐1 gel enhanced the ability of these NSCs‐EVs to promote neurogenesis and neurological functional recovery. The present study demonstrated that IGF‐1‐gel treatment is a promising strategy for improving neurogenesis and neurological functional recovery following SCI by not only promoting NSC differentiation into neurons and oligodendrocytes but also enhancing the bioactivation of EVs derived from grafted NSCs (**Scheme** **1**).

## 2 Results and Discussion

### 2.1 Bioactivity of the IGF‐1 Hydrogel with NSCs

**Figure 1:**
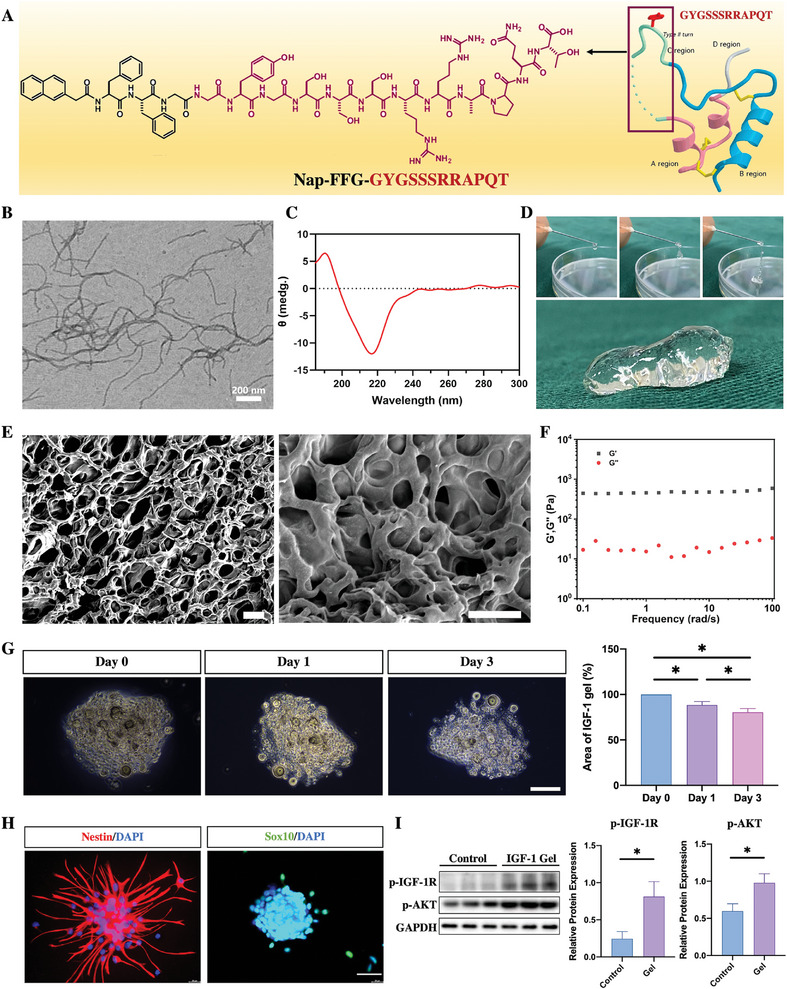
Characteristics of the IGF‐1 gel. A) The chemical structure of the IGF‐1 bioactive supramolecular nanofibers. B) TEM image of IGF‐1 bioactive supramolecular nanofibers. C) CD spectra of the IGF‐1 bioactive supramolecular nanofibers (scale bar, 200 nm). D) Optical images of the IGF‐1 gels at room temperature. E) SEM images of IGF‐1 gels (scale bar 10 µm). F) Rheological properties of IGF‐1 gels. G) The size of the IGF‐1 hydrogel after culture in PBS for 3 days (*n* = 5; scale bar, 100 µm). H) Identification of NSCs with the markers Nestin and Sox 10. I) Western blot results showing that the IGF‐1 gels increased the expression of p‐IGF‐1R and p‐AKT in NSCs (*n* = 3).

**Figure 2:**
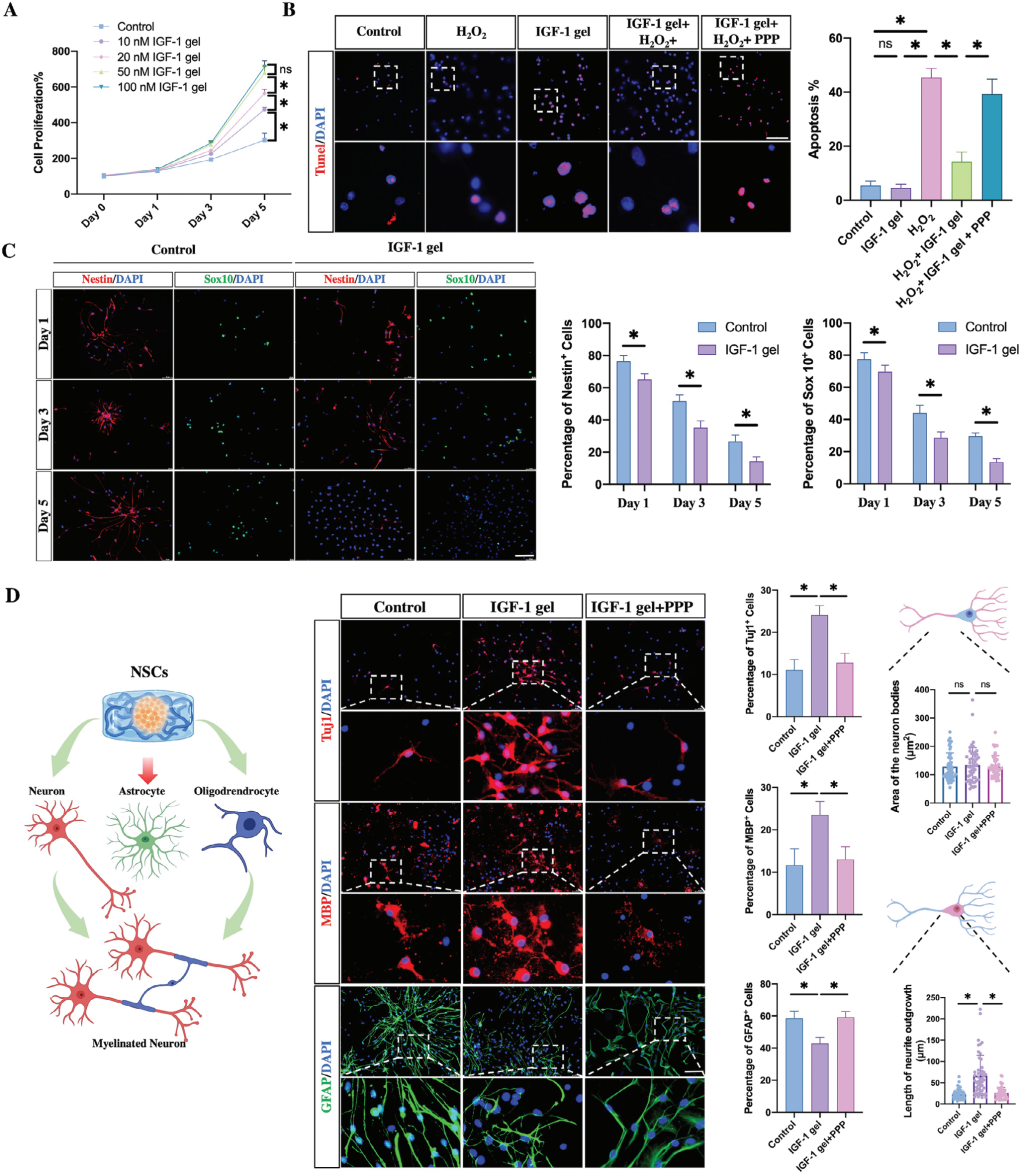
The biological effects of the IGF‐1 gel on NSCs. A) CCK‐8 results showing the proliferation of NSCs cultured with different concentrations of the IGF‐1 gel at different time points (*n* = 5). B) TUNEL staining was used to detect the apoptosis of NSCs cultured with or without IGF‐1‐containing gels after exposure to 100 µm H_2_O_2_ or 100 µM H_2_O_2_ and picropodophyllin for 2 h (*n* = 3; scale bar, 100 µm). C) Immunostaining for Nestin and Sox 10 in NSCs cultured with IGF‐1 gels on days 1, 3, and 5 (*n* = 5; scale bar, 100 µm). D) Assessment of the differentiation of NSCs in IGF‐1 gel with or without picropodophyllin by calculating the percentage of Tuj1‐, MBP‐, and GFAP‐positive cells after 5 days in culture. The area of the neuron bodies and the length of neurite outgrowth were detected in the Tuj‐positive neurons (*n* = 5; scale bar, 100 µm). All the data are presented as the means ± SDs. ^*^, *p* < 0.05; ns, *p* > 0.05.

Previous studies have reported that the C‐region, the amino acid sequence of which is GYGSSSRRAPQT, appears to play a key role in activating IGF‐1R.^[^ ^18c^ ^]^ Therefore, a self‐assembling peptide (Nap‐FFG) was attached to the N‐terminus of IGF‐1C to synthesize an IGF‐1 molecule (**Figure** **1**A). Transmission electron microscopy (TEM) images revealed that the nanofibers formed and became entangled with each other (Figure 1B). The circular dichroism (CD) spectrum showed a positive peak at 190 nm and a negative band centered at 220 nm (Figure 1C). The results of the TEM images and CD spectra were consistent with our previous study^43^. As the injectability of hydrogels is a critical characteristic for applications in the treatment of spinal cord injury, the injectability of the IGF‐1 hydrogel was assessed at room temperature. As shown in Figure 1D, well‐defined droplets extruded well through the needle without any blockage. Subsequently, the microstructure of the IGF‐1 hydrogel was investigated by scanning electron microscopy (SEM), which showed that the IGF‐1 hydrogel exhibited a porous hydrogel structure (Figure 1E). The mechanical properties of the IGF‐1 gel were assessed by dynamic oscillatory rheology measurements. The storage moduli (*G′*) of the IGF‐1 hydrogels approached 500 Pa and were significantly greater than the loss moduli (*G″*) over an angular frequency range of 1–100 Hz, suggesting good stability of the IGF‐1 hydrogel (Figure 1F). When this IGF‐1 hydrogel was kept in PBS for 3 days, the size of the IGF‐1 hydrogel decreased gradually, while the main structure was still preserved (Figure 1G). To evaluate the biological effects of the IGF‐1 hydrogel on NSCs, NSCs were obtained from the subventricular zone of Sprague–Dawley rats and identified through immunostaining with the markers Nestin and SRY‐box containing gene 10 (Sox 10) (Figure 1H). Next, to investigate whether this IGF‐1 hydrogel could activate the IGF signaling pathway in NSCs via its bioactive peptides, the expression of phosphorylated IGF‐1R in NSCs was detected by western blotting. In addition, as IGF has been shown to regulate stem cell bioactivities by upregulating PI3K/Akt signaling,^[^ ^20^ ^]^ phosphorylated Akt (p‐Akt) was detected in NSCs after 1 day of culture on an IGF‐1 gel. As expected, coculture with IGF‐1 gels upregulated both p‐IGF‐R and p‐Akt expression compared with that in NSCs cultured with DMEM/F12 (control) (Figure 1I). To evaluate whether the proliferation of NSCs could be enhanced by treatment with the IGF‐1 gel, NSCs were exposed to culture media supplemented with different concentrations of IGF‐1 (from 10 to 100 nm), and proliferation was detected via cell counting kit‐8 (CCK‐8) assays on days 1, 3 and 5 postculture. Compared with that in the control group, the proliferation of cultured NSCs in the IGF‐1 gel group was markedly increased, especially on days 3 and 5 (**Figure** **2**A). The proliferation of NSCs reached a maximum in the 50 and 100 nm IGF‐1 gel groups, but no difference was noted between these 2 groups (Figure 2A). Therefore, the 50 nm IGF‐1 gel was selected for subsequent experiments.

Overproduction of reactive oxygen species (ROS) following SCI can induce oxidative stress and cytotoxic inflammation, leading to extensive death of nerve cells at lesion sites.^[^ ^21^ ^]^ To evaluate whether the IGF‐1 gel could protect NSCs from ROS‐induced damage, ROS generation was mimicked by adding hydrogen peroxide (H_2_O_2_), which could induce the death of nerve cells by repressing PI3K/AKT signaling.^[^ ^22^ ^]^ The neural spheres were digested into single cells, and TUNEL staining was used to determine the proportion of apoptotic cells. Less than 10% of the cells in the control group and less than 10% of the NSCs were cultured with the IGF‐1 gel (Figure 2B). Exposure of NSCs to 100 µM H_2_O_2_ for 2 h led to an increase in the proportion of apoptotic cells of ≈45%. In contrast, IGF‐1 gel addition markedly reduced the percentage of apoptotic cells to 14% (Figure 2B). Since the picropodophyllin (PPP) has been proven to specifically prevent tyrosine‐phosphorylated IGF‐1R, NSCs were cultured with the PPP in the presence of IGF‐1‐gel and H_2_O_2_ to investigate whether IGF‐1 inhibitors could repress IGF‐1‐1 gel‐induced antiapoptotic effects. The addition of PPP abolished the IGF‐1 gel‐induced antiapoptotic effects, resulting in an increase in the proportion of apoptotic cells (Figure 2B). These data indicate that the IGF‐1 gel could protect NSCs against H_2_O_2_‐induced apoptosis in vitro by activating the IGF signaling pathway, which in turn upregulated PI3K/AKT signaling.

To evaluate whether the IGF‐1 gel could affect the maturation of NSCs, neurospheres were digested into single cells and cultured with or without the IGF‐1 gel. Immunostaining was performed on days 1, 3, and 5 using Nestin and Sox 10. The expression of Nestin and Sox 10 decreased gradually as the NSCs matured, and the proportion of Nestin^+^ and Sox‐10^+^ cells reached a minimum on day 5 postculture. In contrast, compared with those in the control groups, the proportions of Nestin+ and Sox‐10+ cells were lower in the IGF‐1 gel‐treated group than in the control group on days 3 and 5, indicating that the IGF‐1 gel enhanced the maturation of NSCs (Figure 2C).

Posttraumatic inflammation can upregulate a large number of axon growth inhibitors, which accumulate at the lesion site.^[^ ^6^, ^23^ ^]^ These inhibitory molecules induce the differentiation of most grafted NSCs toward astrocytes, leading to the formation of an astrocytic boundary, which causes the failure of neuronal and axonal regrowth.^[^ ^6^, ^23^ ^]^ Therefore, another challenge for cell transplantation, especially for NSCs, is to regulate the differentiation of grafted stem cells and promote their differentiation into neurons and oligodendrocytes in vivo. To evaluate the effect of the IGF‐1 gel on the differentiation of NSCs, NSCs were digested into single cells and cultured for five days with or without the IGF‐1 gel. Immunostaining revealed that IGF‐1 gel treatment increased the proportion of myelin basic protein (MBP)^+^ oligodendrocytes and neuron‐specific class III beta‐tubulin (Tuj‐1)^+^ neurons and decreased the proportion of glial fibrillary acidic protein (GFAP)‐positive astrocytes (Figure 2D). In addition, the length of neurite outgrowth was also increased by the addition of IGF‐1 gel, while no difference was found in the area of the neuronal bodies between the two groups (Figure 2D). To investigate whether IGF‐1 inhibitors could repress IGF‐1‐gel‐induced regulation of NSC differentiation, NSCs were cultured with PPP in the presence of IGF‐1 gels. As anticipated, the PPP considerably increased the percentage of astrocytes while significantly decreasing the proportion of oligodendrocytes and neurons and the length of neurite outgrowth (Figure 2D). These findings suggested that IGF‐1R activation was responsible for the effects of the IGF‐1 gel on NSC differentiation.

### 2.2 Biocompatibility and Biodegradability of the IGF‐1 Gel

**Figure 3:**
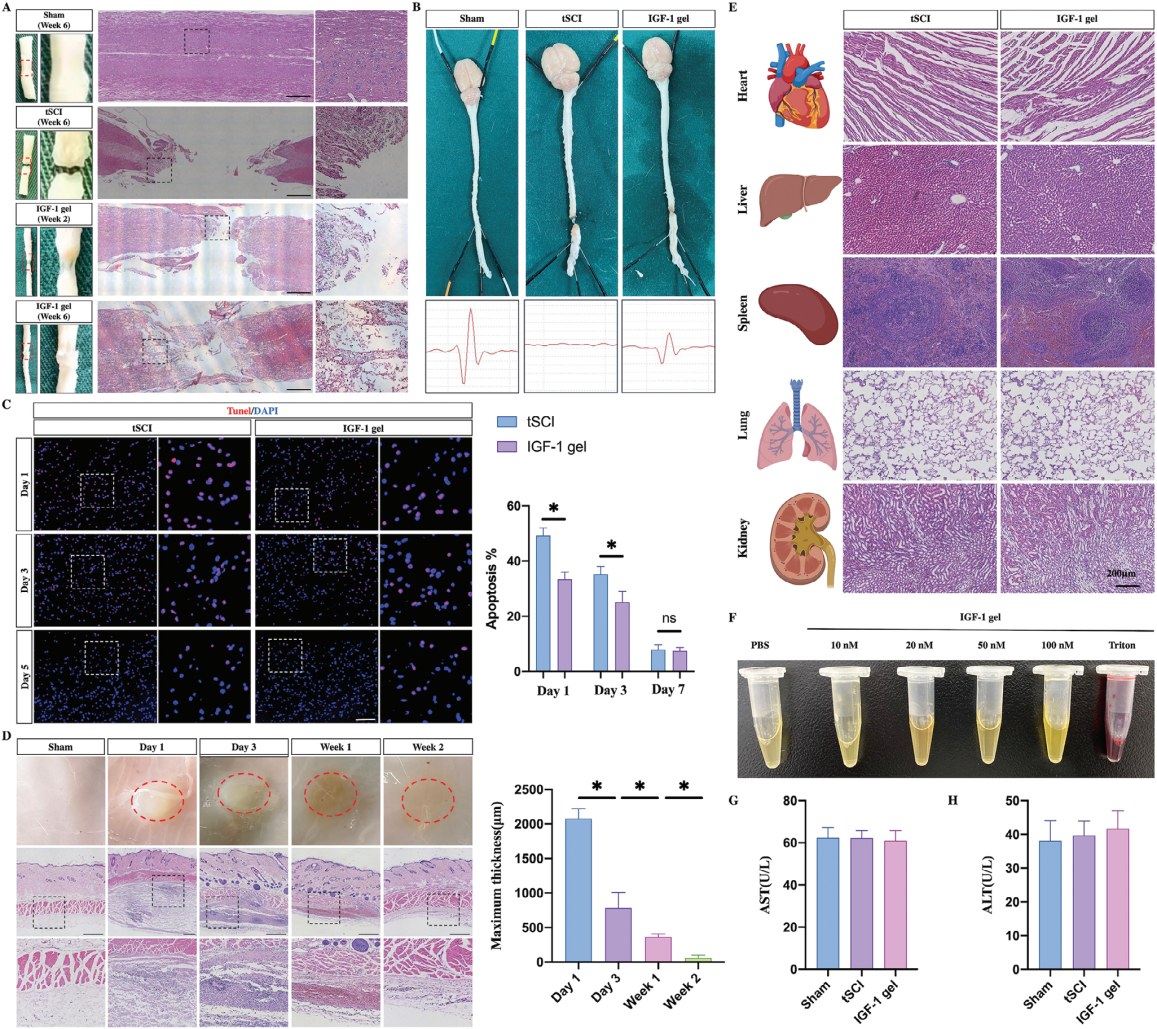
Biodegradability and biocompatibility of IGF‐1 gels. A) General view (left panel) and hematoxylin–eosin (HE) staining of the total transection spinal cord injury (tSCI) model with or without the implantation of IGF‐1 gels (scale bar 500 µm). B) Detection of electrical signaling transmission in the isolated spinal cord. C) TUNEL staining showing apoptosis in the regions adjacent to the transected segment or the implanted IGF‐1 gel (*n* = 5; scale bar, 100 µm). D) After subcutaneous implantation, general observation and HE staining were performed to assess the biodegradability of the IGF‐1 gels, and the results showed that the implanted gels were completely degraded after 2 weeks (*n* = 3, scale bar = 500 µm). E) HE staining of the major organs revealed no pathological abnormalities; the presence of gels; or degraded fragments in the heart, liver, spleen, lung, or kidney (scale bar = 200 µm). F) Photograph of serum extracted from whole blood cocultured with IGF‐1 gels. G,H) The aspartate aminotransferase (AST) and alanine aminotransferase (ALT) levels did not significantly differ between the tSCI rats with and without IGF‐1 gel implantation (*n* = 3). All the data are presented as the means ± SDs. ^*^, *p* < 0.05; ns, *p* > 0.05.

To test the biocompatibility and biodegradability of the IGF‐1 gel, a transected spinal cord injury (tSCI) model was created by completely removing a 3–5 mm segment of the spinal cord at the T8–10 level. IGF‐1 gels were then implanted into the lesion sites. HE staining was performed at 2 and 6 weeks postinjury to observe extracellular matrix features. In the control tSCI rats, the rostral and caudal spinal cords were separated, and no continuity was found in the transected lesion sites (**Figure** **3**A). In contrast, at week 2 postinjury, the tSCI rats with IGF‐1 gel implants had formed a “bridge” across the lesion site to connect the rostral and caudal spinal cord. Hematoxylin‐eosin (HE) staining revealed that the regenerated tissues extended to the lesion sites (Figure 3A). At week 6 postinjury, a connection between the rostral and caudal spinal cords was formed, and HE staining revealed that the tissues were not only generated at the lesion sites but also crossed the transected segment and connected the rostral and caudal areas (Figure 3A). Furthermore, to evaluate whether the IGF‐1 gel could restore electrical signaling transmission within the spinal cord, intact spinal cords were isolated. Compared to that in the sham group, the electrical signal transmission in the tSCI group vanished below the injury site (Figure 3B). In contrast, the transplantation of IGF‐1 gels partly restored electrical signaling transmission (Figure 3B).

To investigate whether the IGF‐1 gel could exert a protective effect on cells adjacent to the injured lesion sites, TdT mediated dUTP nick end labeling (TUNEL) staining was performed on days 1, 3, and 7 postinjury. The rate of apoptosis in the areas adjacent to the transected sites was significantly reduced by the implantation of IGF‐1 gels on days 1 and 3 postinjury. No difference was observed in the percentage of apoptotic cells on day 7 (Figure 3C). These findings indicated that the IGF‐1 gel could protect nerve cells adjacent to lesion sites from apoptosis.

The IGF‐1 gel was implanted subcutaneously to further test its biodegradability. Following gel injection, HE staining showed subcutaneous accumulation, and a large number of inflammatory cells migrated into the injected gels (Figure 3D). Moreover, the average gel thickness decreased with time, and by two weeks after subcutaneous injection, the IGF‐1 gel was completely degraded. In addition, after the implantation of IGF‐1 gels, HE staining of the major organs revealed no pathological abnormalities; IGF‐1 gel; or degraded fragments in the heart, liver, spleen, lung, or kidney (Figure 3E). In addition, serum was extracted from whole blood and cultured on an IGF‐1 gel. The results showed that the different concentrations of IGF‐1 in the gel cocultures produced a clear yellow color similar to that of the PBS control group, while the color of the Triton‐100X group was bright red, indicating hematolysis (Figure 3F). The systemic toxicity of the IGF‐1 gel was tested, and the aspartate aminotransferase (AST) and alanine aminotransferase (ALT) levels did not differ between the tSCI and IGF‐1 gel‐implanted tSCI rats, indicating that no systemic toxicity was induced by the implantation of the IGF‐1 gels (Figure 3G,H).

### 2.3 IGF‐1 Gels Containing NSCs Promote Neuronal Regeneration, Neurite Outgrowth, and Myelin Sheath Formation Following SCI

**Figure 4:**
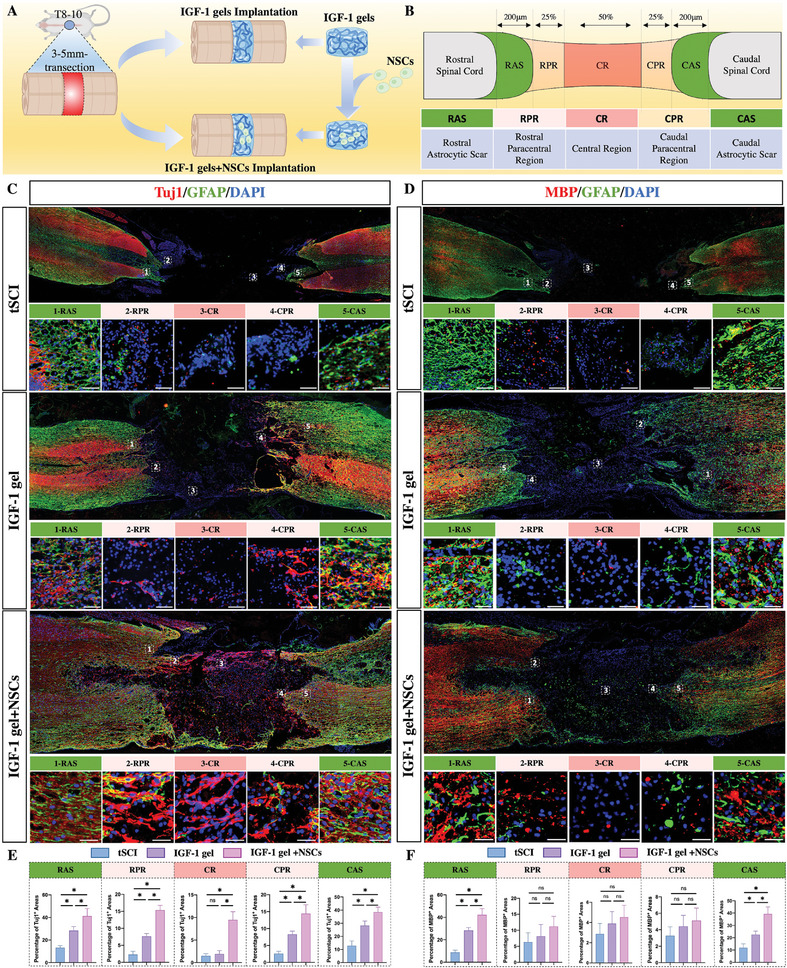
IGF‐1‐stained gels harboring NSCs promote neurite outgrowth and regeneration of the myelin sheath. A) Illustration of the tSCI site and the IGF‐1 gel implant. B) Illustration of the different histological regions that develop following tSCI. C,E) Tuj1 and GFAP double immunostaining in the tSCI, IGF‐1 implantation, and IGF‐1+NSC implantation groups at week 6 following tSCI. Implantation of IGF‐1 gels harboring NSCs increased the number of Tuj1‐positive cells and prolonged the outgrowth of neuronal fibers into the lesion core (*n* = 5, scale bar 100 µm). D,F) MBP and GFAP double immunostaining revealed increased regeneration of myelin sheaths within the astrocytic boundary induced by the NSC‐loaded IGF‐1 gels (*n* = 5; scale bar, 100 µm).

To assess whether IGF‐1 gels could improve neuronal regeneration and neurological functional recovery, IGF‐1 gels were implanted into lesion sites following tSCI (**Figure** **4**A). The regeneration of neurons and myelin sheets was quantified by immunostaining for Tuj1 and MBP at week 6 post‐SCI. In tSCI rats, GFAP‐positive astrocytic scar boundaries formed around the cavity. Moreover, within these astrocytic scars, Tuj1‐positive neurons or MBP‐positive oligodendrocytes were seldom found, indicating that the axons/neurons died from the lesion centers and exhibited a poor capacity for neuronal and axonal regeneration following SCI. In contrast, the rats that received the IGF‐1 gel implants had a significant increase in the density of neurons and myelin sheets within the astrocytic scars adjacent to the cavity (Figure 4C,D). In addition, longitudinally oriented Tuj1‐positive fibers sprouting from neurons regenerated through the glial scar and extended into the cavity (Figure 4C). To compare the percentages of Tuj1‐positive and BMP‐positive cells in different regions, the lesion sites were divided into five regions (Figure 4B): the rostral astrocytic scar (RAS), rostral paracentral region (RPR), central region (CR), caudal paracentral region (CPR), and caudal astrocytic scar (CAS). The percentage of Tuj1‐positive cells increased at the RAS, RPR, CPR, and CAS, while no difference was noted at the CR (Figure 4C–E). Moreover, an increase in the MBP‐positive proportion was noted only in the RAS and CAS (Figure 4D–F). These findings showed that implantation of the IGF‐1 gel enhanced neuronal regeneration and outgrowth. However, the capacity to improve myelin sheet regrowth was observed only within the astrocytic boundary.

To evaluate whether the IGF‐1 gel‐loaded NSCs had an enhanced effect on improving neuronal and axonal regrowth, NSCs were pelleted, mixed with the IGF‐1 gel, and subsequently implanted into lesion sites (Figure 4A). Immunostaining revealed that Tuj1‐positive neurite outgrowth was significantly improved. These neuronal fibers not only grew across the astrocytic boundary and extended into the cavity but also formed a “bridge” that reconnected the two ends of the injury site (Figure 4C). In addition, many Tuj1‐positive cells were noted in the CR, indicating regeneration of neurons in the center of the cavity (Figure 4C). In agreement with these findings, when compared to those of tSCI rats and tSCI rats that received only IGF‐1 gel implantation, the tSCI rats that had both IGF‐1 gel and NSC implantation had the highest percentage of Tuj1^+^ areas in all five regions (Figure 4E). However, when the proportion of MBP^+^ areas was calculated, there was no difference in the RPR, CPR, or CR, although MBP‐positive oligodendrocytes have the potential to regenerate through the astrocytic boundary and extend into the cavity (Figure 4D). The increase in percentage was confirmed to be statistically significant only for the RAS and CAS (Figure 4F).

**Figure 5:**
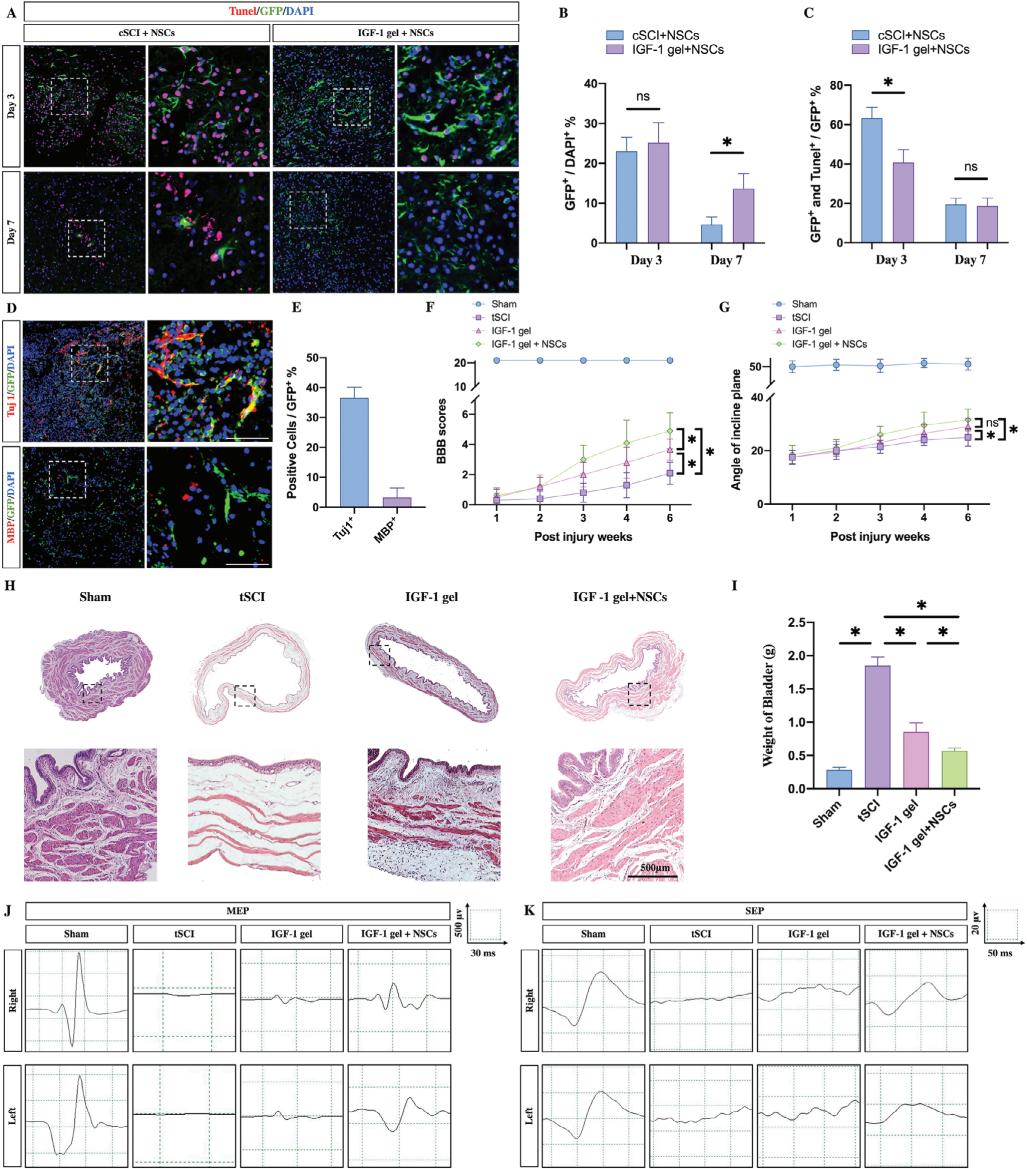
The IGF‐1 gel promoted neurological recovery by promoting grafted NSC differentiation into neurons. A–C) IGF‐1 gel treatment protected the grafted GFP‐positive NSCs from apoptosis on day 3 and day 7 postinjury (*n* = 5; scale bar, 100 µm; ^*^, *p* < 0.05; ns, *p* > 0.05). D,E) The IGF‐1 gel‐generated NSCs (GFP‐positive cells) were more likely to differentiate into neurons (GFP and Tuj1 double‐positive cells) at lesion sites than into oligodendrocytes (GFP and MBP double‐positive cells) at week 6 postinjury (*n* = 5, scale bar 100 µm; ^*^, *p* < 0.05; ns, *p* > 0.05). F,G) Evaluation of neurological recovery by the BBB score and the inclined‐plane test (*n* = 10). H) HE staining showing muscle fiber bundles in the bladder wall at week 6 following tSCI (scale bar, 500 µm). I) Assessment of bladder weight at week 6 posttSCI (*n* = 5). All the data are presented as the means ± SDs. ^*^, *p* < 0.05; ns, *p* > 0.05. J,K) Images of right/left hindlimb MEPs and SEPs in tSCI, IGF‐1 gel‐implanted, and IGF‐1 gel + NSC‐implanted rats at week 6 postinjury.

To further investigate whether the IGF‐1 gel could protect NSCs from apoptosis and whether the regeneration of neurite outgrowth and myelin sheaths was associated with the differentiation of the grafted NSCs, we used green fluorescent protein (GFP) to label the grafted NSCs and transplanted the GFP‐tracked NSCs with the IGF‐1 gel. To compare apoptosis with the direct transplantation of NSCs, a weight‐drop contusion model of SCI (cSCI) was used, and GFP‐labeled NSCs were injected into the lesion sites. TUNEL staining was subsequently performed on days 3 and 7 postinjury to evaluate the percentage of apoptotic cells. On day 3 postinjury, no difference was found in the percentage of GFP‐positive cells between the rats that received NSCs only and those that received IGF‐1 gel+NSCs. However, the apoptosis rate was significantly greater in the cSCI rats that received direct NSC injection (GFP and TUNEL double‐positive cells; **Figure** **5**A–C). On day 7, the rats that received IGF‐1 gel and NSC implantation had a markedly greater percentage of GFP‐positive cells, while no difference in GFP or TUNEL double‐positive cells was noted between these 2 groups (Figure 5A–C). These results indicated that the IGF‐1 gels protected the graft NSCs from apoptosis in the early period after SCI. To evaluate whether these GFP‐positive NSCs could differentiate into neurons and oligodendrocytes, immunostaining was performed at week 6 postinjury. Nearly 25% of the GFP‐positive cells were found in the lesion sites (Figure 5D). Moreover, ≈10% of the cells were Tuj1 and GFP double‐positive, accounting for 30% of the GFP‐positive NSCs (Figure 5D,E). However, few MBP and GFP double‐positive cells were found in these areas (Figure 5D,E). These results indicated that grafted NSCs are more likely to differentiate into neurons in vivo than oligodendrocytes in the presence of the IGF‐1 gel. This finding explained why coimplantation of IGF‐1 gel and NSCs increased the number of Tuj1‐positive cells but did not increase the number of MBP‐positive oligodendrocytes within lesion sites. Taken together, the IGF‐1 gel provides a favorable environment for the survival and differentiation of implanted NSCs, and these NSCs could form a “relay station” to connect the neuronal fibers originating from the intrinsic neurons in the astrocytic scar regions on either side.

Neurological function was also evaluated. Basso, Beattie, and Bresnahan test (BBB) scores were calculated, and inclined‐plane tests were performed to assess hindlimb locomotor function. In the tSCI rats, the BBB score was ≤2. The average scores improved to nearly 3.6 in the rats that had received IGF‐1 gel implants. In contrast, in the IGF‐1 gel+NSC‐implanted rats, the BBB score increased to 5 (Figure 5F; additional videos are included in Supporting Information). Consistent with the BBB scores, the inclined‐plane tests showed that the IGF‐1 gel+NSC‐implanted rats had greater angles than did the other two groups (Figure 5G). The BBB and inclined‐plane results indicated that IGF‐1 gel implantation combined with NSCs had the strongest effect on improving the locomotor function of the hind limb. In addition, the urinary system was evaluated by HE staining, and the weight of the bladders was assessed. HE staining revealed that the muscle bundles under the bladder wall were markedly thinned following tSCI. In contrast, the thickness of these muscle bundles was increased by the implantation of IGF‐1 gel or IGF‐1 gel+NSCs (Figure 5H). Assessment of bladder weight revealed that it was significantly increased at week 6 following tSCI. In contrast, the implantation of IGF‐1 gels or IGF‐1 gel+NSCs repressed this increase in the weight of the bladders, and the lowest weight was noted in the IGF‐1 gel+NSC‐implanted rats (Figure 5I). These data indicate that the function of the bladder was recovered by IGF‐1 gel+NSC implantation. Electrophysiology revealed that, compared to that of tSCI rats, the implantation of IGF‐1 gels or IGF‐1 gel+NSCs could partly restore the waveforms of motor‐evoked potentials (MEPs) (Figure 5J) and somatosensory‐evoked potentials (SEPs) (Figure 5K) in the left/right hindlimb at week 6 postinjury.

### 2.4 The Combined Implantation of IGF‐1 Gels and NSC‐Derived Extracellular Vesicles (EVs) Promotes Neuronal Outgrowth and Neurological Recovery

When comparing neurogenesis at the lesion edge between the rats implanted with the gel and the rats implanted with IGF‐1+NSCs, the rats that received both IGF‐1 gel and NSC implantation showed a marked outgrowth of Tuj1‐positive fibers that broke through the astrocytic boundary. Neurite outgrowth in IGF‐1 gel‐implanted rats was significantly shorter than that in gel+NSC‐implanted rats. Moreover, these outgrowing fibers were more likely to originate from the nerve cells within the astrocytic scar neighboring the cavity than from the implanted NSCs in the gels. Additionally, compared with IGF‐1 gel implantation, IGF‐1 gel+NSC implantation had a greater effect on increasing the MBP‐positive areas within both the RAS and CAS. These results indicated that the implantation of NSCs might promote the regeneration of neurite outgrowth and myelin sheaths within the edge of lesion sites through long‐distance communication. Since EVs play a key role in long‐distance cell‒cell communication^[^ ^7^, ^8^ ^]^ and following neurotrauma, endogenous NSCs are activated and contribute to neuronal and axonal repair,^[^ ^3^, ^24^ ^]^ it was hypothesized that by releasing EVs, implanted NSCs might communicate with endogenous NSCs to promote neurite outgrowth and myelin sheath regeneration within the edge of lesion sites.

**Figure 6:**
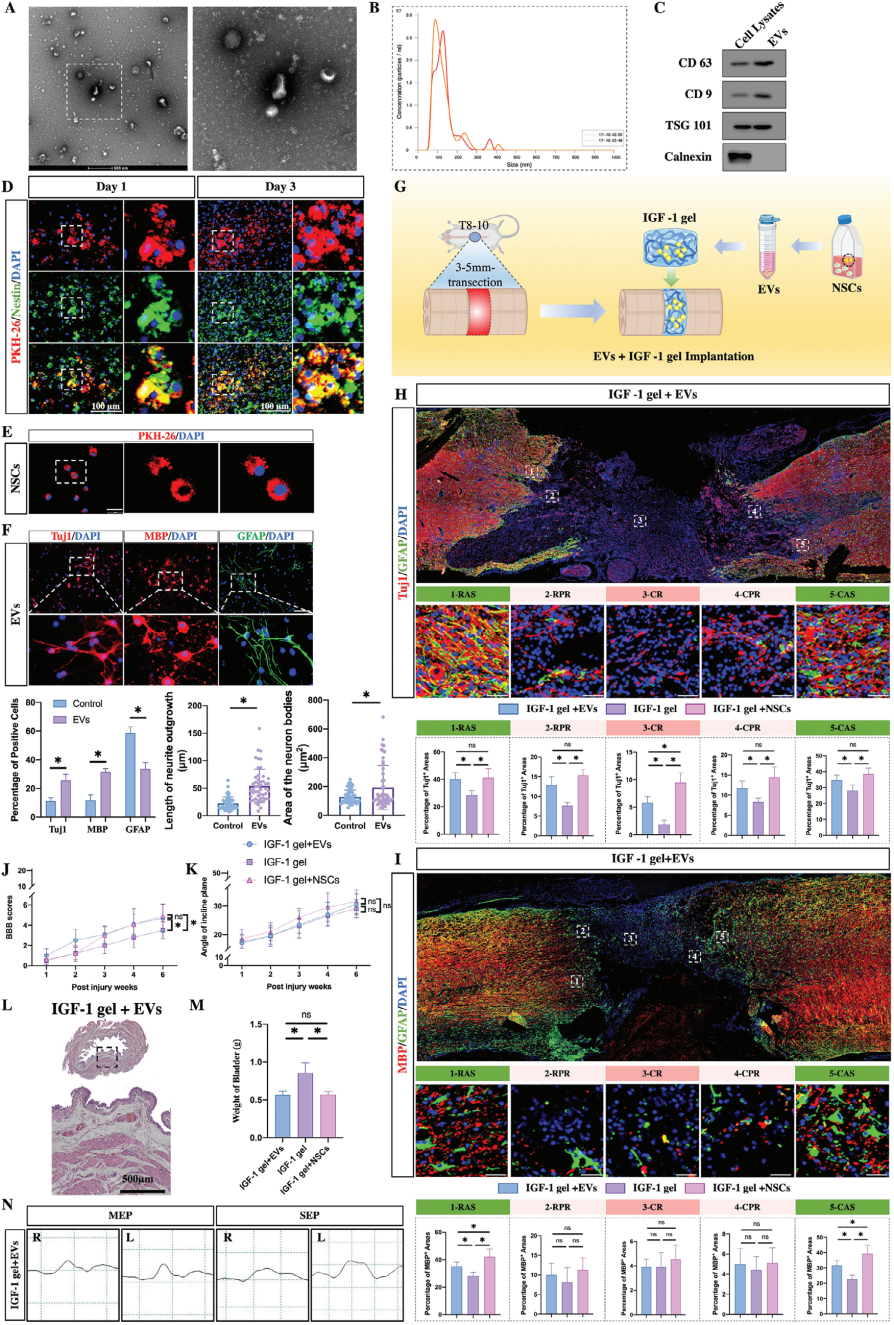
IGF‐1 gels loaded with extracellular vesicles (EVs) shed from NSCs (NSC‐EVs) improve neurological recovery by regulating the differentiation of endogenous NSCs. A–C) Identification of NSC‐EVs by A) transmission electron microscopy, B) dynamic light scattering, and C) western blotting. D) Immunostaining showing that Nestin+NSCs were colabeled with PKH‐26 from labeled NSC‐EVs on days 1 and 3 postimplantation of IGF‐1 gel+NSC‐EVs (scale bar, 100 µm). E) PKH‐26‐labeled NSC‐EVs were clearly detected in the cytoplasm of NSCs (scale bar, 100 µm). F) Immunostaining showing that NSC‐EVs promote the differentiation of NSCs into neurons and oligodendrocytes in vitro (*n* = 5; scale bar, 100 µm). G) Illustration of IGF‐1 gel+NSC‐EV implantation. H) Immunostaining showing that IGF‐1‐expressing gels containing NSC‐EVs enhanced neuronal regeneration and neurite outgrowth at week 6 postinjury (*n* = 5, scale bar 100 µm). I) Immunostaining showing that the number of MBP‐positive oligodendrocytes at the astrocytic boundary was increased at week 6 postinjury (*n* = 5; scale bar, 100 µm). J,K) Assessment of neurological recovery in terms of BBB scores and the inclined‐plane test (*n* = 10). L) HE staining showing muscle fiber bundles in the bladder wall at week 6 following tSCI (scale bar, 500 µm). M) Assessment of bladder weights at week 6 postinjury (*n* = 5). All the data are presented as the means ± SDs. ^*^, *p* < 0.05; ns, *p* > 0.05. N) Images of right/left hindlimb MEPs and SEPs in IGF‐1 gel + EV‐implanted rats at week 6 postinjury.

To confirm this hypothesis, EVs derived from NSCs were first collected and identified (**Figure** **6**A–C). PKH‐26 was used to label EVs, and these PKH‐26‐labeled EVs were loaded into IGF‐1 gels and implanted into lesion sites in tSCI rats. Nestin was used to mark endogenous NSCs at the edge of the lesion sites on days 1 and 3 postinjury. Following SCI, an increasing number of nestin‐positive endogenous NSCs migrated to and accumulated around the lesion sites. Moreover, many of the EVs were colabeled with PKH‐26 (Figure 6D), indicating that the EVs loaded into the IGF‐1 gels could migrate out of the implants to the edge of the lesion site and be taken up by endogenous NSCs. These data indicated that by migrating into endogenous NSCs, the EVs within the IGF‐1 gel promoted the extension of neurite outgrowth fibers into injured lesion sites.

To confirm this possibility, EVs labeled with PKH‐26 were incubated with NSCs. After 24 h of culture, we noticed that the NSCs had PKH‐26‐labeled EVs (red) in their cytoplasm or around their nuclei (Figure 6E), confirming that the EVs were adopted by the NSCs. Next, the bioeffects of EVs on NSC differentiation were evaluated in vitro by immunostaining. After 5 days of culture, the percentage of neurons and oligodendrocytes was much greater in the NSCs exposed to EVs than in the control cells (Figure 6F). Moreover, further morphological observation revealed that the presence of EVs lengthened outgrowing neurites and extended the regions of neuronal somata (Figure 6F). EV‐loaded IGF‐1 gels were then implanted into the lesion sites in vivo (Figure 6G). Immunostaining for Tuj1 revealed that long‐distance Tuj1 neuronal fibers broke through the glial boundary and extended into the cavity (Figure 6H). Quantification of the Tuj1‐positive areas showed that, compared to those of gel‐implanted rats, the percentage of Tuj1‐positive areas in all five regions was greater in the rats that received EVs+IGF‐1 gel implants (Figure 6H). No differences were found in the RAS, RPR, CPR, or CAS between the NSC+IGF‐1 gel‐implanted rats and the control rats. However, in the CR, the proportion of Tuj1^+^ areas in the rats that received EVs+IGF‐1 gel was lower than that in the NSCs+IGF‐1 gel‐implanted rats. In addition, compared with rats implanted with the gel alone, a greater percentage of the EV+IGF‐1 gel‐implanted rats had MBP‐positive areas in the RAS and CAS but a lower proportion of MBP‐positive areas in the RAS and CAS than did the rats implanted with the NSCs+IGF‐1 gels (Figure 6I). No differences were found in the other regions (Figure 6I). These data indicated that EVs carried by IGF‐1 gels promoted neurite outgrowth and myelin sheath regeneration by regulating endogenous NSC differentiation. It is noteworthy that the EVs derived from NSCs exhibited anti‐apoptotic and neuroprotective effects in various pathological conditions,^[^ ^25^ ^]^ and the IGF‐1 gels were able to upregulate IGF‐1 signaling pathway in the nerve cells closing to the lesion sites, these alterations could protect the neurons and oligodendrocytes from death. Therefore, the increase in Tuj1‐ and MBP‐positive areas was due not only to endogenous differentiation but also to the decrease in the death of neurons and oligodendrocytes.

Neurological function assessment revealed that, compared to those of the gel‐implanted rats, the BBB scores of the EV+IGF‐1 gel‐implanted rats were improved, but these improvements were less pronounced than those of the NSC gel‐implanted rats (Figure 6J; the videos are included in Supporting Information). Among the three groups, there were no variations in the inclined‐plane test angle (Figure 6K). HE staining of the bladder revealed that atrophy of the muscle bundles was ameliorated by treatment with EV‐loaded gels (Figure 6L). Assessment of bladder weight showed that, compared to rats implanted with gel alone, the EV+IGF‐1 gel‐implanted rats had reduced bladder weights. However, no difference in bladder weight was noted compared to that in the NSC+IGF‐1 gel‐implanted group (Figure 6M). Electrophysiology revealed that the waveforms of the MEP and SEP were partly recovered in the left/right hindlimb by implantation of the EV‐loaded IGF‐1 gels at week 6 postinjury (Figure 6N).

EVs constitute a significant portion of the secretome, which facilitates paracrine function and permits regulatory function advantages. Moreover, they could avoid the drawbacks of direct stem cell implantation.^[^ ^7^ ^]^ Therefore, the use of EVs from different sources of stem cells has emerged as a promising clinical therapeutic approach for treating SCI. In the present study, the histological results showed that the implantation of NSC‐loaded IGF‐1 gels formed a Tuj1‐positive “relay station” in the core lesion site, leading to a significant increase in the number of Tuj1‐positive areas in the CR when compared to the implantation of EV‐loaded IGF‐1 gels. In addition, the implantation of NSC‐loaded IGF‐1 gels also revealed a greater percentage of MBP‐positive areas in the RAS and CAS than did the implantation of EV‐loaded IGF‐1 gels. These data indicated that, in a stable and favorable environment, compared with EVs, grafted NSCs exerted enhanced effects on neurogenesis. Therefore, the combination of biomaterials is critical for cell transplantation for the clinical treatment of SCI.

### 2.5 IGF‐1 Stimulation Enhances the Biological Effects of Total EVs (tEVs) Derived from NSCs During NSC Differentiation

**Figure 7:**
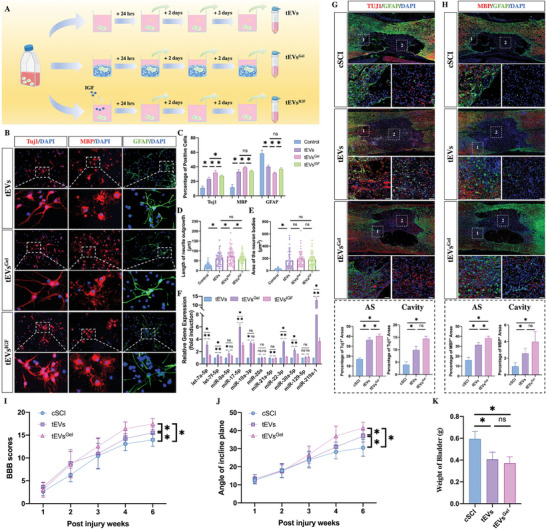
IGF‐1 gels enhance the bioactivity of tEVs. A) Illustration of the collection of tEVs, tEVs^Gel^, and tEVs^IGF^. B–E) Immunostaining demonstrating that tEVs^Gel^ is more effective than tEVs or tEVs^IGF^ at inducing NSC differentiation into neurons and oligodendrocytes (*n* = 5; scale bar, 100 µm). F) The IGF‐1 gels upregulated miRNAs related to axonal regeneration and remyelination within the tEVs (*n* = 3). All the data are presented as the means ± SDs. ^*^, *p* < 0.05; ns, *p* > 0.05. G,H) Injection of tEVs^Gel^ promoted neuron and oligodendrocyte regeneration at week 6 postinjury (*n* = 5; scale bar, 100 µm). I,J) BBB and inclined‐plane scores of cSCI, tEV‐injected, and tEV^Gel^‐injected rats (*n* = 10). K) Assessment of bladder weights at week 6 postinjury (*n* = 5).

Recent studies have shown that the bioactivity and biological cargo of EVs can respond to stimuli and the surrounding microenvironment.^[^ ^9^ ^]^ For example, upon capsaicin stimulation, neurons release EVs enriched with miR‐21, which in turn induces neuropathic hypersensitivity by promoting macrophage polarization toward the proinflammatory M1 phenotype.^[^ ^9c^ ^]^ Similarly, an inflammatory environment promotes astrocyte polarization toward toxic “A1” reactive astrocytes, which release many types of proinflammatory cytokines that can damage adjacent nerve cells.^[^ ^26^ ^]^ In contrast, an ischemic environment can transform astrocytes toward the protective “A2” reactive astrocyte type, which secretes various neurotrophic factors that promote neuronal and axonal regeneration.^[^ ^27^ ^]^ In addition, as the grafted NSCs differentiated into nerve cells in a short time, the EVs in our system were derived not only from the grafted NSCs but also from the nerve cells differentiated from the grafted NSCs. Therefore, to investigate whether IGF‐1‐containing gels affect the bioeffects and cargo of EVs released by NSCs and NSC‐derived nerve cells, EVs were collected from differentiating NSCs alone on days 1, 3, and 5 postculture and subsequently combined with the tEV preparation. The EVs from the NSCs cultured with IGF‐1 gels were collected on days 1, 3, and 5 and combined as the tEV^Gel^ preparation (**Figure** **7**A). To investigate whether the biological effect of tEVs could be affected by the IGF‐1 gel, NSCs were cultured for 5 days with the addition of tEVs or tEVs^Gel^. As expected, tEVs increased the percentages of neurons and oligodendrocytes. Moreover, compared to tEVs, tEVs^Gel^ resulted in a greater percentage of neurons and oligodendrocytes, thus displaying an even greater effect on NSC differentiation (Figure 7B–E). In addition, the length of neurite outgrowth was significantly increased by ^tEVGel^, but no difference was noted between tEV‐treated NSCs and those treated with ^tEVGel^ in the areas of the neuronal somata (Figure 7B–E). To investigate whether the IGF‐1 gel affects the bioactive cargoes within tEVs, several remyelination‐related and axon regeneration‐related miRNAs^[^ ^28^ ^]^ were detected in tEVs cultured with or without IGF‐1 gels. PCR analysis revealed that most tEV miRNAs were increased following culture on an IGF‐1 gel (Figure 7F). In particular, miR‐219 was upregulated more than tenfold, and miR‐17‐5p was increased fivefold.

To further compare the effects of IGF‐1 gels and growth factor‐IGF on the bioeffects and cargo of tEVs, 10 ng mL^−1^ IGF was added to NSCs for 24 h culture. EVs were collected on days 1, 3, and 5 postculture and combined with tEVs^IGF^ (Figure 7A). To investigate the effects of tEVs^IGF^ on NSC differentiation, NSCs were cultured with tEVs^IGF^ for 5 days. As expected, tEVs^IGF^ had a stronger biofunction effect than tEVs, resulting in a greater percentage of neurons and a lower percentage of astrocytes (Figure 7B–E). However, compared to that of tEVs^Gel^, the bioeffect of tEVs^IGF^ was attenuated, resulting in a decrease in the percentage of neurons and oligodendrocytes and an increase in the proportion of astrocytes (Figure 7B–E). The detection of remyelination‐related and axon regeneration‐related miRNAs within tEVs^IGF^ showed that although several miRNAs were upregulated by treatment with IGF, the degree of these IGF‐induced upregulations was weakened when compared to tEVs^Gel^‐treated NSCs (Figure 7F).

To determine the effects of tEVs in vivo, a weight‐drop contusion model of SCI (cSCI) was used. In this model, the dura remains intact following spinal cord injury, preventing leakage of injected tEVs. The in vivo effect of tEVs was investigated in the absence of IGF‐1 gels. To transplant the tEVs, a 3‐day continuous intrathecal injection was administered to the cSCI rats. Six weeks postcSCI, a cavity was observed at the lesion site, and an astrocytic scar (AS) had formed surrounding this cavity (Figure 7G,H). In the AS region, the number of Tuj1‐positive and MBP‐positive cells was markedly reduced (Figure 7G,H). In tEV‐treated rats, regenerated neuronal fibers passed through the astrocytic boundary and extended into the cavity, resulting in increased expression in both Tuj1‐positive and MBP‐positive areas in the AS and cavity. In contrast, the rats injected with tEVs^Gel^ had a longer distance of regenerated neurite outgrowth in the cavity and a greater percentage of neurons and oligodendrocytes in the AS (Figure 7G,H). However, compared to that in tEV‐treated rats, the percentage of neurons and oligodendrocytes in the cavity was not greater in the tEVGel‐injected group (Figure 7G,H). According to the neurological test results, compared to those of control cSCI rats and tEV‐injected rats, the rats that received tEVs^Gel^ had the highest BBB scores (Figure 7I) and inclined plane angles (Figure 7J), which was consistent with the histological results. Although the bladder weight was reduced by the injection of either tEVs or tEVs^Gel^, there was no difference between the 2 groups (Figure 7K). These data showed that the IGF‐1 gels could directly modify the miRNAs within EVs and that these alterations in miRNAs within tEVs contributed to the enhanced biological activity of these tEVs in the mediation of NSC differentiation and improved neurite outgrowth and myelin sheath regeneration following cSCI.

### 2.6 IGF‐1 Gels Enriched Axon Regeneration‐Related and Remyelination‐Related miRNAs in tEVs Released from Nerve Cells Differentiated from NSCs in an Inflammatory Environment

**Figure 8:**
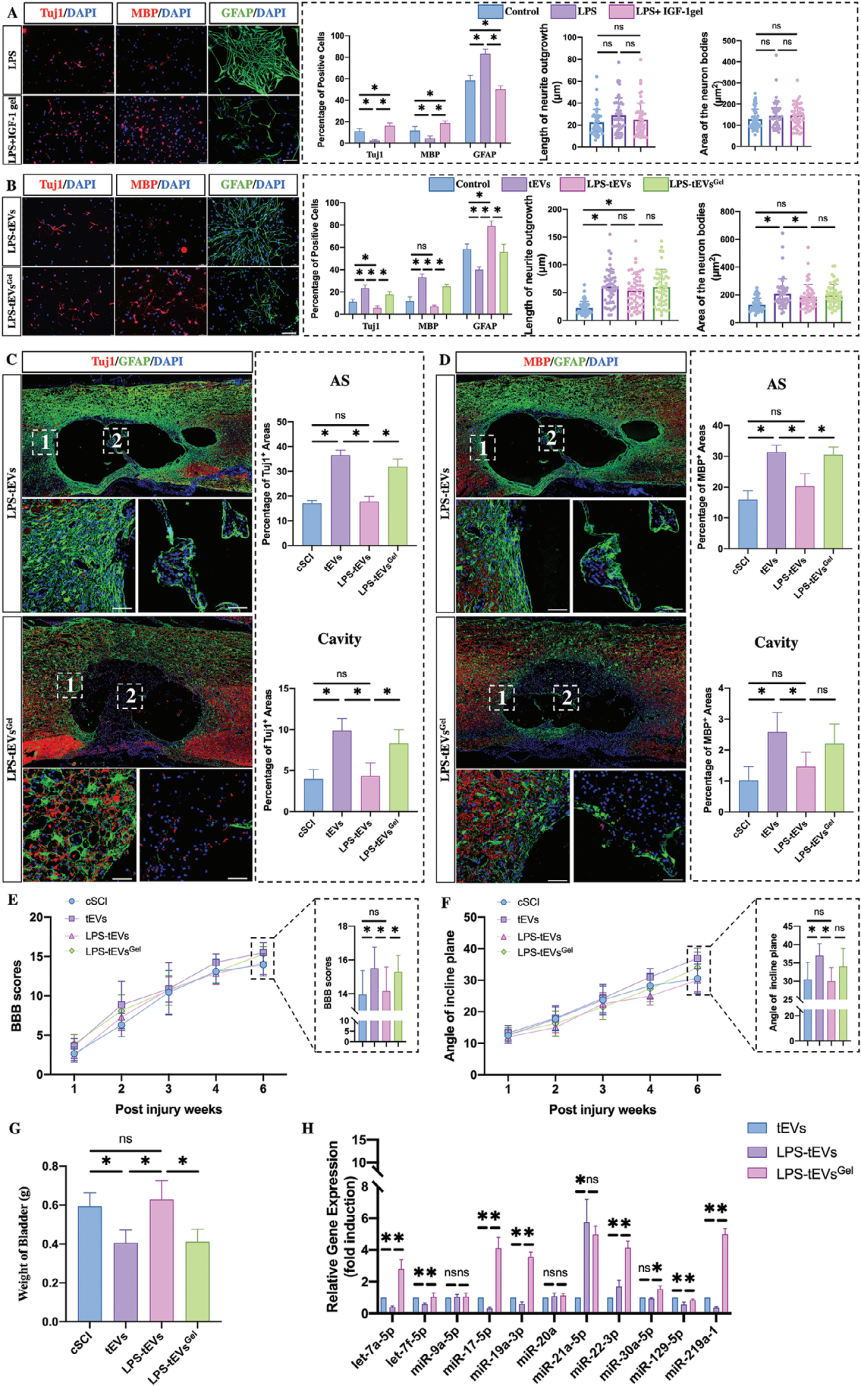
IGF‐1 gels inhibit the adverse effects of inflammation on the bioactivity of tEVs. A) LPS stimulation induces NSC differentiation into astrocytes, while IGF‐1 gel treatment abolishes this effect (*n* = 5; scale bar, 100 µm). B) LPS‐tEVs induce NSC differentiation into astrocytes but reduce the percentage of neurons and oligodendrocytes. LPS‐tEVs^Gel^ did not have a negative effect on LPS efficacy. (*n* = 5, scale bar 100 µm). C,D) Injection of LPS‐tEVs inhibited the regeneration of neurons and myelin sheaths, while LPS‐tEVs^Gel^ increased the Tuj1‐ and MBP‐positive areas in the astrocytic boundary and cavity (*n* = 5, scale bar 100 µm). E,F) BBB and inclined‐plane scores of rats that received injections of LPS‐tEVs or LPS‐tEVs^Gel^ (*n* = 10). G) Assessment of bladder weights at week 6 postinjury (*n* = 5). H) Detection of miRNAs related to axonal regeneration and remyelination, showing that LPS stimulation decreases the expression of these miRNAs in tEVs derived from differentiated NSCs. IGF‐1 gels can prevent these decreases, leading to the relative upregulation of these beneficial miRNAs (*n* = 3). All the data are presented as the means ± SDs. ^*^, *p* < 0.05; ns, *p* > 0.05.

Following SCI, various primary pathophysiological alterations can directly activate inflammation, which, in turn, triggers a series of complex cellular and molecular interactions directly affecting the bioactivities of nerve cells.^[^ ^23^, ^29^ ^]^ To investigate whether the IGF‐1 gel can protect NSCs in inflammatory environments, the inflammatory environment was mimicked in vitro by adding lipopolysaccharides (LPS) to differentiating NSCs. This significantly increased the percentage of astrocytes produced, with a reduction in neurons and oligodendrocytes. In contrast, the IGF‐1 gel abolished the LPS‐induced increase in differentiation into astrocytes, leading to an increase in the proportion of neurons and oligodendrocytes. Moreover, the addition of LPS did not affect the length of neurite outgrowth or the area of the neuronal somata (**Figure** **8**A).

Next, to investigate whether the biological activities of tEVs derived from NSCs and from nerve cells differentiated from NSCs could be modified by inflammatory stimulation, tEVs were collected from LPS‐treated NSCs during differentiation and compounded as LPS‐tEVs, and tEVs from LPS‐treated NSCs cultured with IGF‐1 gels were collected as LPS‐^tEVGels^. These tEVs were subsequently added to NSCs to determine their effects on regulating the differentiation of NSCs. Compared with tEV‐treated NSCs, LPS‐tEVs lost their ability to promote neuronal and oligodendroglial differentiation, resulting in a marked increase in the percentage of astrocytes. In contrast, LPS‐tEVs^Gel^ partly reversed these effects, increasing the fraction of neurons and oligodendrocytes (Figure 8B).

To confirm the effects of these tEVs in vivo, cSCI rats were injected with LPS‐tEVs or LPS‐tEVs^Gel^. The percentage of Tuj1‐positive and MBP‐positive cells within the AS and cavity was calculated, and the results showed that, compared with those in tEV‐treated rats, the percentage of Tuj1‐positive and MBP‐positive areas was significantly lower in the LPS‐tEV‐treated group (Figure 8C,D). In contrast, the percentage of Tuj1^+^ and MBP^+^ areas within the AS and cavity was greater in the LPS‐tEV gel‐injected rats than in both the cSCI rats and the LPS‐tEV‐injected rats. The results of the BBB and inclined‐plane tests were worse for the LPS‐tEV‐injected rats than for the tEV‐injected rats, which was consistent with the histological data, and there was no difference between the LPS‐tEV‐injected rats and the cSCI rats (Figure 8E,F). In contrast, injection of LPS‐tEVs^Gel^ continued to affect the biology of the EVs, leading to improvements in BBB scores and inclined‐plane test results (Figure 7E,F). Similar results were found for bladder weight (Figure 8G).

PCR was used to assess miRNA expression in tEVs, and the results showed that the addition of LPS decreased the expression of miRNAs related to remyelination and axonal regeneration in EVs (Figure 8H). In contrast, the expression of several of these miRNAs increased after the addition of the IGF‐1 gel (Figure 8H). In particular, the levels of let‐7a, miR‐17, miR‐19, miR‐22, and miR‐219 were significantly reduced in the LPS‐tEVs and markedly elevated in the LPS‐tEVs^Gel^. Taken together, these data suggested that in the inflammatory environment, the beneficial biological effects of EVs were inhibited due to the downregulation of miRNAs related to axonal regeneration and remyelination. However, the IGF‐1 gel protected NSCs against this inflammatory inhibition, leading to the upregulation of these beneficial miRNAs.

Emerging evidence has shown that the beneficial biological effects of grafted stem cells are closely related to their paracrine activity.^[^ ^30^ ^]^ In the present study, the data revealed that an unfavorable environment induced the loss of the beneficial effects of EVs derived from grafted NSCs. In contrast, these EV‐induced effects could be enhanced in a favorable environment. Therefore, for future clinical application, the implementation of biomaterials could lead to a stable and advantageous 3D environment for improving the bioactivity of EVs derived from grafted cells and enhancing beneficial “cell‒cell” communication with autologous nerve cells at lesion sites.

## 3 Conclusion

Here, the application of IGF‐1 gels activated the IGF‐1 downstream signaling pathway and provided a favorable 3D environment. It enhanced NSC proliferation, prevented cell apoptosis, and promoted the differentiation of the grafted NSCs into neurons and oligodendrocytes. In addition, the IGF‐1 gels enriched the beneficial miRNAs shed by the grafted NSCs or their differentiated progeny. These EVs migrated out of the implanted IGF‐1 gels and formed long‐distance cell‒cell contacts with autologous endogenous NSCs. Thus, by improving the biological function of grafted NSCs and their released EVs, the combined implantation of NSCs and IGF‐1 gels significantly promoted neurite outgrowth and myelin sheath regeneration following SCI. These data indicate that the use of supramolecular nanofiber hydrogels bound to IGF‐1 bioactive peptides is a promising therapeutic strategy for carrying exogenous NSCs to treat SCI.

## 4 Experimental Section

### NSC Culture and Differentiation

NSCs were isolated as described in the previous study.^[^ ^6a,c^ ^]^ Briefly, cells were obtained from the subventricular zone and cultured as suspended neurospheres for 7 days in NSC culture medium supplemented with (all from Gibco, MA, USA) serum‐free DMEM/F12; B27, 2%; epidermal growth factor (EGF), 20 ng mL^−1^; and basic fibroblast growth factor (bFGF), 10 ng mL^−1^. Half of the culture medium was replaced with fresh NSC culture medium every three days. After 7 days of suspension culture, the neurospheres were digested into single cells and cultured on poly‐D‐lysine‐coated coverslips at a density of 1 × 105 cells per well. The digested single NSCs were adherent and grown in DMEM/F12 and 2% B27‐containing NSC differentiation media. The medium was changed every 3 days.

### EV Collection and Tracking

To obtain NSC‐derived EVs, cultured neurospheres were harvested after 7 days and washed three times with PBS. These neurospheres were then resuspended in DMEM/F12 medium for an additional 24 h. The supernatants were collected as NSC‐conditioned media. To collect EVs during the process of NSC differentiation, neurospheres were digested into single cells that were adherent and cultured in an NSC differentiation medium with or without an IGF‐1 gel. To simulate the inflammatory environment, adherent NSCs were treated with 10 ng mL^−1^ LPS with or without the IGF‐1 gel. The supernatants from these groups were harvested on days 1, 3, and 5 postculture. Concentrated EVs were obtained as previously described.^[^ ^6b,c^ ^]^ Briefly, to remove cell debris, the collected conditioned medium or supernatant was centrifuged at 300 × g for 10 min, 2000 × g for 20 min, and 10 000 × g for 45 min at 4 °C. The EVs were then harvested by centrifugation at 1 00 000 × g at 4 °C for 90 min. The harvested EVs were dissolved in 100 µL of PBS and stored at −80 °C. Transmission electron microscopy (TEM), dynamic light scattering (DLS), and western blotting for TSG 101, CD9, CD63, and calnexin were performed to identify the EVs.

PKH26 (Sigma‒Aldrich) was used to label the EVs according to the manufacturer's instructions. Briefly, the EVs were incubated with 4 L of PKH‐26 diluted in 1 mL of Diluent C for 4 min at room temperature. After that, the EVs were collected by centrifugation at 1 00 000 × g for 70 min after being diluted in 1 mL of PBS.

### Preparation of the IGF‐1 Gel

The IGF‐1 mimetic compound 1 was prepared as previously described.^[^ ^18c^ ^]^ Briefly, standard solid‐phase peptide synthesis (SPPS) was used to create all peptide derivatives by using 2‐chlorotrityl chloride resin and the corresponding N‐Fmoc‐protected amino acids with proper side chains. PBS (pH 7.4) was used to dissolve the compounds in a suspension at a concentration of 0.5 weight percent. GelMA was synthesized as previously described.^[^ ^31^ ^]^ At a concentration of 8.5 mm, the photoinitiator LAP (lithium phenyl‐2,4,6 trimethylbenzoylphosphinate) was added to gelatin methacryloyl. The IGF‐1 bioactive supramolecular nanofiber suspension (1 mmol L^−1^) was heated to transparency and then diluted into a gelatin methacryloyl solution to a concentration of 10 µmol L^−1^. Various concentrations of the IGF‐1 gel were further diluted from this stock solution. Transmission electron microscopy (TEM) and circular dichroism (CD) spectroscopy were performed as described in a previous study.^[^ ^18c^ ^]^

### SEM Images

The hydrogels were placed in a freeze drier after being frozen at −20 °C. To maintain their original internal cross‐linked structure, the hydrogel samples were flash‐frozen using liquid nitrogen. The samples were subsequently sputter‐coated with platinum (Pt) for 60 s. At an accelerating voltage of 10 kV, morphological observations were made using an SEM.

### Rheological Experiments

A rotary rheometer was used to measure the rheological parameters. At a constant strain of 1%, an angular frequency sweep (0.1–100%) was conducted. The lower plate of the machine was filled with 1 mL of each hydrogel and heated to 37 °C. Next, the upper plate was lowered to a gap of 1 mm to start measuring the rheological parameters.

### Cell Proliferation and Apoptosis Assays

A Cell Counting Kit‐8 (CCK‐8; Beyotime, China) was used to quantify the cell number. NSCs were digested into single cells and seeded at a density of 1 × 103 per well. An IGF‐1 gel was used to culture the cells at various concentrations (10, 20, 50, and 100 nm). Ten microliters of CCK‐8 solution was added to each well. On days 1, 3, and 5, the optical density (OD) values at 450 nm were tested for the different groups.

To induce the overproduction of reactive oxygen species (ROS), single NSCs were seeded at a density of 1 × 105 per well with or without IGF‐1 gel and incubated with a medium containing 100 µM H_2_O_2_ for 2 h. A TUNEL test kit (Beyotime, China) was used to calculate apoptotic rates following the instructions provided by the manufacturer.

### Immunofluorescence Staining

To identify NSCs, NSC neurospheres were cultured on poly D‐lysine‐coated coverslips for 24 h and immunostained for Nestin and Sox10. To evaluate the maturity of the NSCs, they were digested into single cells, mixed with IGF‐1 gels, and plated in culture plates; on days 1, 3, and 5, these NSCs were immunostained with Nestin and Sox 10. For the control groups, digested single NSCs were seeded on poly D‐lysine‐coated coverslips. To test the differentiative capacity of NSCs, single NSCs were cultured under the following conditions after being seeded at a density of 1 × 105 per well: 2.5 mL NSC differentiation medium (control); 2.5 mL NSC differentiation medium+IGF‐1 gel; 2.5 mL NSC differentiation medium+IGF‐1 gel+1 µm picropodophyllin (PPP); 2.5 mL NSC differentiation medium+25 µL NSC‐EVs; 2.5 mL NSC differentiation medium+25 µL total EVs (tEVs, EVs released by NSCs and NSC‐derived nerve cells); 2.5 mL NSC differentiation medium+25 µL tEVs^Gel^; 2.5 mL NSC differentiation medium+10 ng mL^−1^ LPS; 2.5 mL NSC differentiation medium+IGF‐1 gel+10 ng mL^−1^ LPS; 2.5 mL NSC differentiation medium+25 µL LPS‐tEVs; and 2.5 mL NSC differentiation medium+25 µL LPS‐tEVs^Gel^. Cells were treated with 4% paraformaldehyde for 24 h after 5 days in culture before being immunostained for glial fibrillary acidic protein (GFAP), neuron‐specific class III beta‐tubulin (Tuj1), and myelin basic protein (MBP). To perform tissue immunofluorescence staining, spinal cords from SCI rats were obtained at week 6 postinjury and treated with 4% paraformaldehyde for 24 h.

To observe the PKH‐26 EVs, the spinal cords were fixed with 4% paraformaldehyde for 24 h and incubated with 10%, 20%, or 30% sucrose solution at 4 °C. Using a Leica RM2135 or CM3050 electric slicer, the spinal cord was divided into longitudinal slices that were 4 m thick and centered on the damage lesion. The sliced tissues were then subjected to immunofluorescence staining.

The primary antibodies used were as follows: rabbit anti‐nestin for NSCs (1:1000; Abcam, United Kingdom), rabbit anti‐SOX 10 for NSCs (1:1000; Abcam, United Kingdom), rabbit anti‐GFAP for astroglia (1:1000; Abcam, United Kingdom), mouse anti‐MBP for oligodendrocytes (1:2000; Abcam, United Kingdom), and rabbit anti‐Tuj1 for neurons (1:1000; Abcam, United Kingdom). Alexa Fluor 488 (green, 1:50; Elabscience, China) and Cy3 (red, 1:50; Elabscience, China) were used as secondary antibodies. Images were observed and photographed using a DM‐6B fluorescence microscope (Leica, Germany) or an Axio Observer fluorescence microscope (Zeiss, Germany). For cell counting, fields with 300–500 cells were chosen at random. ImageJ was used to calculate the percentage of positive cells in vitro and the percentage of positive regions in vivo.

### Spinal Cord Injury

Female Sprague‒Dawley rats aged 8–10 weeks (weight ≈250 g) were selected for the transected spinal cord injury (tSCI) model and the weight‐drop contusion SCI (cSCI) model. All rats were kept in a facility with a 12‐h light/dark cycle and were regulated for temperature and humidity. The Anhui Medical University Ethics Committee granted approval for animal treatments (LLSC 20210674) under the terms of the 2000 Edinburgh revision of the Declaration of Helsinki. Briefly, the rats were anesthetized with sodium pentobarbital (35 mg kg^−1^). For tSCI, a 3 mm segment of the spinal cord was completely removed at the T9–10 level. The tSCI rats were then randomly divided into four groups: those that received no treatment (SCI), those that received IGF‐1 gel implantation, those that received IGF‐1 gels containing 1 × 106 NSCs, and those that received IGF‐1 gels containing 75 µL of EVs. For cSCI, a weight‐drop injury was induced at the T9–10 level. Twenty‐five microliters of saline, 25 µL of tEVs, 25 µL of tEVs^Gel^, 25 µL of LPS‐tEVs, or 25 µL of LPS‐tEVs^Gel^ were administered to cSCI rats via a 3‐day continuous intrathecal injection. The neurological outcomes were assessed by the BBB open‐field test and the inclined plane test at different time points by two independent individuals. Heart, liver, spleen, lung, and kidney tissues were also collected, and hematoxylin–eosin (H&E) staining was performed at week 1 postinjury. Bladders were collected at week 6 postinjury and stained with H&E.

### Electrophysiological Examinations

To record the electrical signals in the spinal cord, transected spinal cords were isolated from the rats at week 6 postinjury. The stimulating electrodes were inserted into the spinal cord above the injury sites, while the recording electrodes were inserted into the spinal cord below the injury sites. Electrophysiological examinations were performed at week 6 postinjury. To record MEPs, the stimulating electrode was implanted into the skin of the head (2.5 mm anterior to bregma, 2.5 mm lateral to the midline, and at a depth of 1 mm), and the recording electrodes were inserted into the gastrocnemius muscle. A ground electrode was inserted into the skin on the back. To record SEPs, the stimulating electrode was inserted into the gastrocnemius muscle, while the recording electrodes were inserted into the skin of the head. Both the stimulation and the recording electrodes were connected to a biological information acquisition device.

### Assessment of the Degradation and Biocompatibility of IGF‐1 Gels

Transected SCI rats were used to evaluate the degradation and biocompatibility of IGF‐1 gels at lesion sites. tSCI rats were randomly divided into sham, tSCI, and gel groups (rats that received IGF‐1 gel implantation). The spinal cords were collected, and H&E staining was performed at 2 and 6 weeks postinjury. To assess the subcutaneous biodegradability of the IGF‐1 gels, 0.5 mL of IGF‐1 was injected into the backs of the rats. The surrounding tissue was collected and fixed in 4% paraformaldehyde for H&E staining on day 1, day 3, week 1, and week 2 after injection.

### RNA Extraction and Quantitative PCR

Following the manufacturer's instructions, RNA was extracted from the cells using an RNA kit (TRIzol, Gibco). cDNA was generated using Superscript III RT Reaction Mix (Invitrogen). The qPCR procedure was carried out using SYBR Green Master Mix (Applied Biosystems). miRNA‐related qRT‒PCR was performed with a Bulge‐LoopTM miRNA qRT‒PCR Starter Kit with their primers (RiboBio, Guangzhou, China), and U6 was used as an internal reference.

### Western Blot Analysis

Cells were lysed on ice in RIPA lysis buffer containing phosphatase inhibitor (Thermo Fisher) and protease inhibitor (Thermo Fisher). A Thermo Fisher's BCA protein assay kit was used to measure the concentrations of the proteins that were collected. SDS‒PAGE was used to separate the collected proteins and the resulting proteins were subsequently transferred to a PVDF membrane. After the membranes were blocked with skim milk at room temperature for 2 h, they were incubated with primary antibodies (p‐IGF‐1R and p‐Akt) overnight at 4 °C. The SuperSignal West Pico‐enhanced chemiluminescence reagent (Thermo Scientific) was used to visualize the blots after the secondary antibody had been applied to the membranes for 1 h, and ImageJ was used to quantify the results.

### Statistical Analysis

Statistical analysis was performed using SPSS software version 16.0 (Chicago, IL, USA) and GraphPad Prism version 8. The data are presented as the mean ± standard deviation. Statistical significance was examined using Student's two‐sample *t*‐test or one‐way analysis of variance (ANOVA) with Tukey's post hoc method. *p* < 0.05 indicated statistical significance.

## Conflict of Interest

The authors declare no conflict of interest.

## Author Contributions

P.S., T.H., and Z.W. contributed equally to this work. C.S. and X.W. contributed to the conception and design of the work; P.S. contributed to the research design, acquisition, and analysis of the data, and manuscript writing; T.H. contributed to the manuscript writing, analysis, and interpretation of the data; P.S., F.H., and Z.W. contributed to the acquisition of the data and software used in the work; and T.H., Z.W., Y.L., and Y.W. contributed to the acquisition and analysis of the data. All the authors have read and approved the final manuscript.

## Supporting information

Supplemental Movie 1

Supplemental Movie 2

Supplemental Movie 3

Supplemental Movie 4

Supplemental Movie 5

## Data Availability

The data that support the findings of this study are available from the corresponding author upon reasonable request.

## References

[1] a) M. A. Anderson , J. W. Squair , M. Gautier , T. H. Hutson , C. Kathe , Q. Barraud , J. Bloch , G. Courtine , Nat. Neurosci. 2022, 25, 1584; 36396975 10.1038/s41593-022-01196-1

[2] a) Z. Shang , M. Wang , B. Zhang , X. Wang , P. Wanyan , BMC Med. 2022, 20, 284; 36058903 10.1186/s12916-022-02482-2 PMC9442938

[3] a) M. Stenudd , H. Sabelström , J. Frisén , JAMA Neurol. 2015, 72, 235; 25531583 10.1001/jamaneurol.2014.2927

[4] S. Ceto , K. J. Sekiguchi , Y. Takashima , A. Nimmerjahn , M. H. Tuszynski , Cell Stem Cell 2020, 27, 430. 32758426 10.1016/j.stem.2020.07.007 PMC7484050

[5] Y. Yang , Y. Fan , H. Zhang , Q. Zhang , Y. Zhao , Z. Xiao , W. Liu , B. Chen , L. Gao , Z. Sun , X. Xue , M. Shu , J. Dai , Biomaterials 2021, 269, 120479. 33223332 10.1016/j.biomaterials.2020.120479

[6] a) P. Song , T. Han , X. Xiang , Y. Wang , H. Fang , Y. Niu , C. Shen , Stem Cell Res. Ther. 2020, 11, 178; 32410702 10.1186/s13287-020-01691-x PMC7227078

[7] a) D. Rufino‐Ramos , P. R. Albuquerque , V. Carmona , R. Perfeito , R. J. Nobre , L. Pereira de Almeida , J. Control Release 2017, 262, 247; 28687495 10.1016/j.jconrel.2017.07.001

[8] a) C. C Bavisotto , F. Scalia , A. Marino Gammazza , D. Carlisi , F. Bucchieri , E. Conway de Macario , A. J. L. Macario , F. Cappello , C. Campanella , Int. J. Mol. Sci. 2019, 20, 434; 30669512 10.3390/ijms20020434 PMC6359416

[9] a) A. Datta Chaudhuri , R. M. Dasgheyb , L. R. DeVine , H. Bi , R. N. Cole , N. J. Haughey , Glia 2020, 68, 128; 31469478 10.1002/glia.23708

[10] T. Führmann , P. N. Anandakumaran , M. S. Shoichet , Adv. Healthcare Mater. 2017, 6, 1130. 10.1002/adhm.201601130 28247563

[11] a) R. Y. Tam , T. Fuehrmann , N. Mitrousis , M. S. Shoichet , Neuropsychopharmacology 2014, 39, 169; 24002187 10.1038/npp.2013.237 PMC3857664

[12] a) L. Fan , C. Liu , X. Chen , Y. Zou , Z. Zhou , C. Lin , G. Tan , L. Zhou , C. Ning , Q. Wang , ACS Appl. Mater. Interfaces 2018, 10, 17742; 29733569 10.1021/acsami.8b05293

[13] M. A. Anderson , T. M. O'Shea , J. E. Burda , Y. Ao , S. L. Barlatey , A. M. Bernstein , J. H. Kim , N. D. James , A. Rogers , B. Kato , A. L. Wollenberg , R. Kawaguchi , G. Coppola , C. Wang , T. J. Deming , Z. He , G. Courtine , M. V. Sofroniew , Nature 2018, 561, 396. 30158698 10.1038/s41586-018-0467-6 PMC6151128

[14] a) X. Hu , R. Li , Y. Wu , Y. Li , X. Zhong , G. Zhang , Y. Kang , S. Liu , L. Xie , J. Ye , J. Xiao , J. Cell. Mol. Med. 2020, 24, 8166; 32515141 10.1111/jcmm.15478 PMC7348165

[15] V. Nieto‐Estévez , Ç. Defterali , C. Vicario‐Abejón , Front. Neurosci. 2016, 10, 52. 26941597 10.3389/fnins.2016.00052 PMC4763060

[16] a) A. N. Ziegler , S. W. Levison , T. L. Wood , Nat. Rev. Endocrinol. 2015, 11, 161; 25445849 10.1038/nrendo.2014.208 PMC5513669

[17] a) S. W. Carlson , K. E. Saatman , J. Neurotrauma. 2018, 35, 1467; 29455576 10.1089/neu.2017.5374 PMC5998830

[18] a) M. J. Webber , J. Tongers , C. J. Newcomb , K. T. Marquardt , J. Bauersachs , D. W. Losordo , S. I. Stupp , Proc. Natl. Acad. Sci. U. S. A. 2011, 108, 13438; 21808036 10.1073/pnas.1016546108 PMC3158182

[19] a) L. D. D'Andrea , G. Iaccarino , R. Fattorusso , D. Sorriento , C. Carannante , D. Capasso , B. Trimarco , C. Pedone , Proc. Natl. Acad. Sci. U. S. A. 2005, 102, 14215; 16186493 10.1073/pnas.0505047102 PMC1242306

[20] a) X. Zhang , L. Zhang , X. Cheng , Y. Guo , X. Sun , G. Chen , H. Li , P. Li , X. Lu , M. Tian , J. Qin , H. Zhou , G. Jin , PLoS One 2014, 9, e113801; 25474202 10.1371/journal.pone.0113801 PMC4256305

[21] a) S. Kumar , T. Theis , M. Tschang , V. Nagaraj , F. Berthiaume , Antioxidants (Basel) 2021, 10, 1013; 34202655 10.3390/antiox10071013 PMC8300734

[22] L. Quintos , I. A. Lee , H. J. Kim , J. S. Lim , J. Park , M. K. Sung , Y. R. Seo , J. S. Kim , Nutr. Res. Pract. 2010, 4, 351. 21103079 10.4162/nrp.2010.4.5.351 PMC2981716

[23] a) T. M. O'Shea , J. E. Burda , M. V. Sofroniew , J. Clin. Invest. 2017, 127, 3259; 28737515 10.1172/JCI90608 PMC5669582

[24] a) B. Brousse , O. Mercier , K. Magalon , F. Daian , P. Durbec , M. Cayre , Stem Cell Rep. 2021, 16, 1792; 10.1016/j.stemcr.2021.05.002 PMC8282429 34087164

[25] a) Q. Liu , Y. Tan , T. Qu , J. Zhang , X. Duan , H. Xu , Y. Mu , H. Ma , F. Wang , Life Sci. 2020, 254, 117772; 32437794 10.1016/j.lfs.2020.117772

[26] O. G. Bhalala , L. Pan , V. Sahni , T. L. McGuire , K. Gruner , W. G. Tourtellotte , J. A. Kessler , J. Neurosci. 2012, 32, 17935. 23238710 10.1523/JNEUROSCI.3860-12.2012 PMC3538038

[27] S. A. Liddelow , B. A. Barres , Immunity 2017, 46, 957. 28636962 10.1016/j.immuni.2017.06.006

[28] a) P. Yuan , L. Ding , H. Chen , Y. Wang , C. Li , S. Zhao , X. Yang , Y. Ma , J. Zhu , X. Qi , Y. Zhang , X. Xia , J. C. Zheng , Front. Cell Dev. Biol. 2021, 9, 601600; 34055767 10.3389/fcell.2021.601600 PMC8155619

[29] A. D. Greenhalgh , S. David , F. C. Bennett , Nat. Rev. Neurosci. 2020, 21, 139. 32042145 10.1038/s41583-020-0263-9

[30] V. Veneruso , F. Rossi , A. Villella , A. Bena , G. Forloni , P. Veglianese , J. Control Release 2019, 300, 141. 30851286 10.1016/j.jconrel.2019.02.038

[31] J. W. Nichol , S. T. Koshy , H. Bae , C. M. Hwang , S. Yamanlar , A. Khademhosseini , Biomaterials 2010, 31, 5536. 20417964 10.1016/j.biomaterials.2010.03.064 PMC2878615

